# Early Transcriptome Signatures from Immunized Mouse Dendritic Cells Predict Late Vaccine-Induced T-Cell Responses

**DOI:** 10.1371/journal.pcbi.1004801

**Published:** 2016-03-21

**Authors:** Nicolas Dérian, Bertrand Bellier, Hang Phuong Pham, Eliza Tsitoura, Dorothea Kazazi, Christophe Huret, Penelope Mavromara, David Klatzmann, Adrien Six

**Affiliations:** 1 Sorbonne Universités, UPMC Univ Paris 06, UMRS 959, Immunology, Immunopathology, Immunotherapy, Paris, France; 2 AP-HP, Clinical Investigation Center in Biotherapy, Hôpital Pitié-Salpêtrière, Paris, France; 3 INSERM, UMRS 959, "Immunology, Immunopathology, Immunotherapy", Paris, France; 4 Molecular Virology Laboratory, Hellenic Pasteur Institute, Athens, Greece; Memorial Sloan-Kettering Cancer Center, UNITED STATES

## Abstract

Systems biology offers promising approaches for identifying response-specific signatures to vaccination and assessing their predictive value. Here, we designed a modelling strategy aiming to predict the quality of late T-cell responses after vaccination from early transcriptome analysis of dendritic cells. Using standardized staining with tetramer, we first quantified antigen-specific T-cell expansion 5 to 10 days after vaccination with one of a set of 41 different vaccine vectors all expressing the same antigen. Hierarchical clustering of the responses defined sets of high and low T cell response inducers. We then compared these responses with the transcriptome of splenic dendritic cells obtained 6 hours after vaccination with the same vectors and produced a *random forest* model capable of predicting the quality of the later antigen-specific T-cell expansion. The model also successfully predicted vector classification as low or strong T-cell response inducers of a novel set of vaccine vectors, based on the early transcriptome results obtained from spleen dendritic cells, whole spleen and even peripheral blood mononuclear cells. Finally, our model developed with mouse datasets also accurately predicted vaccine efficacy from literature-mined human datasets.

## Author Summary

Vaccines are designed to elicit effective immune responses against antigens. The various vector platforms used in vaccine development are diverse and complex, rendering the selection of promising vaccines vector challenging. We have designed a modeling strategy that predicts the propensity of vaccine vectors to elicit strong late T-cell responses using transcriptome material obtained 6 hours after vaccination. Our model, designed with mouse datasets, also predicted vector efficacy from mined human data. Thus, molecular signatures obtained 6 hours after vaccination can predict vaccine efficacy at 2 weeks post vaccination, which should help in vaccine development.

## Introduction

The development of vaccines against complex chronic diseases such as HIV or cancer has been largely unsuccessful so far. Novel vaccine technologies are rationally designed to generate appropriate protective immune responses [1], notably efficient T-cell responses. Such vaccine vectors include plasmid DNA, viral and bacterial vectors, and virus-like particles (VLPs). The intrinsic characteristics of these vectors, including their capacity to stimulate innate immunity and to activate and target the antigen to antigen-presenting cells, determine in large part their immunogenicity and thus their potency as vaccine or gene therapy vectors [2–4]. However the rational design of vectors is limited by various aspects, such as the partial understanding of the factors governing the induction of optimal immunity (i.e. the activation of the innate immune system by various vector components, the effect upon adaptive immunity…) or the possible dependence of vector efficacy on the specificity of the target diseases.

Systems biology has been introduced in vaccine development to assist in circumventing these limitations and shorten the vaccine development process. Systems biology may not only help to better understand, analyze and reconstruct the complex immune interactions between the pathogen/vaccine and host immune system, but may also improve the *in silico* testing models for vaccine candidates. Systems biology approaches have proven capable to predict immune responses induced after vaccination [5,6]. For example, expression patterns of genes associated with the efficient processing of peptides for major histocompatibility complex presentation have been identified as useful surrogate markers of vaccine efficacy, obviating the need to perform challenge studies [7]. Signatures derived from antibody repertoire profiling on peptide microarrays during the natural course of influenza infection were shown to be predictive of the efficacy of influenza vaccines [8]. Multivariate analysis performed on human peripheral blood mononuclear cell (PBMC) microarray data, obtained 3 days after vaccination, identified innate immune response–related signatures that predicted the late adaptive immune response to the YF-17D yellow fever vaccine [9].

In this manuscript, we describe a methodology that enabled us to successfully predict the adaptive immune responses induced by large sets of vaccine vectors of different classes, ranging from infectious particles to VLPs and DNA. All these vectors expressed the same antigen, the immune response to which was measured using a validated standardized method. We developed our model based on the analysis of transcriptomic data, obtained 6 hours after vaccination, that could predict the antigen-specific immune responses induced at the peak of the response, 5–10 days later. It is noteworthy that this model, developed in mice, successfully predicted vaccine-induced responses from literature-mined human datasets.

## Results

### Vaccine vector classification according to antigen-specific T-cell expansion

**Fig 1:**
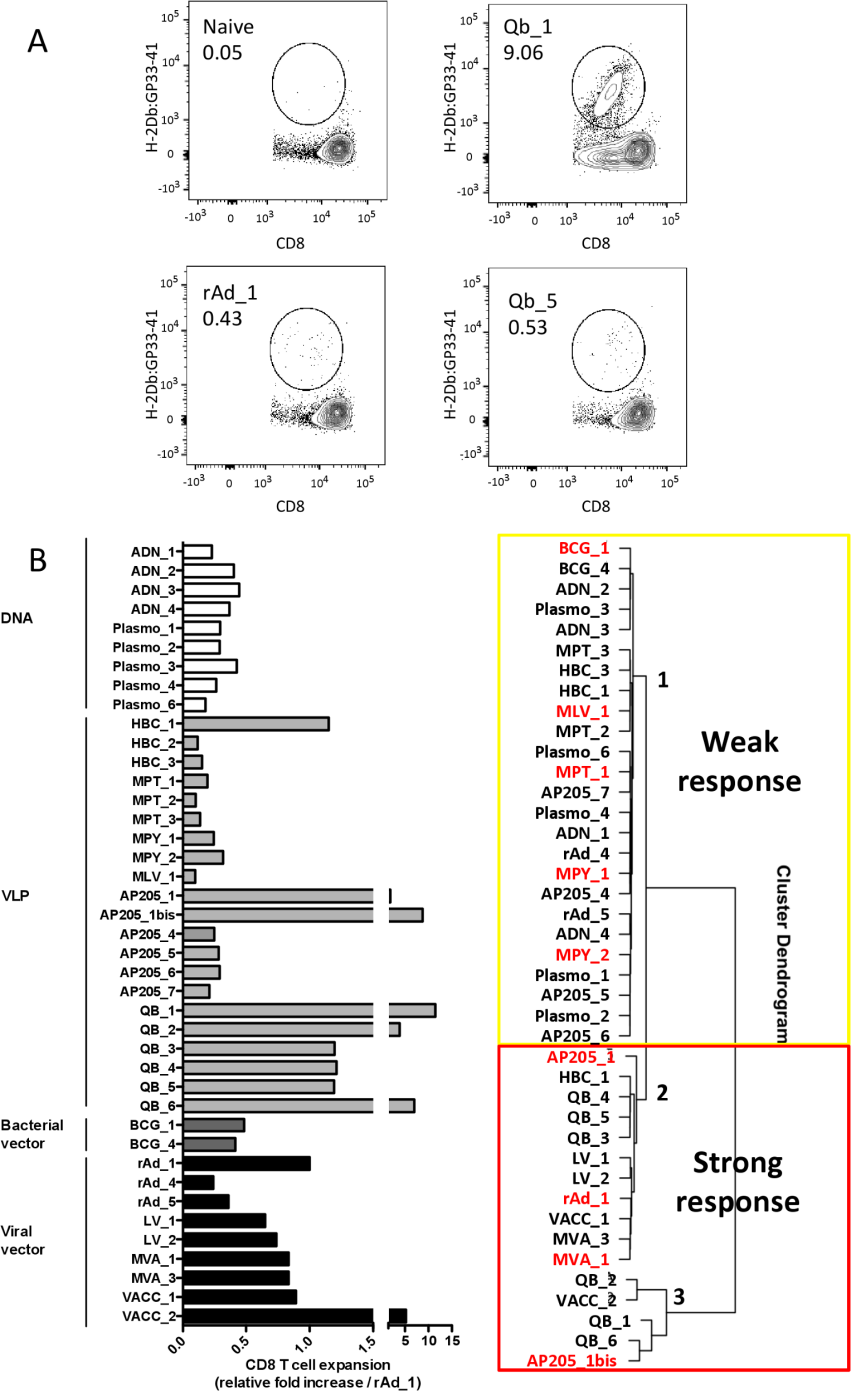
LCMV gp33-41 model antigen-expressing/displaying vector T-cell response analysis. A. Evaluation of gp33-41 specific T-cell frequency in mice immunized with Qb_1 or Qb_5 VLPs, with rAd_1 and control naive mice, by H-2Db:gp33-41 tetramer staining. B. For each vector tested, gp33-41 antigen-specific responses were evaluated at days 5, 7 and 10 in groups of 3–5 vaccinated mice. The normalized “CD8 T-cell expansion” value was calculated as the average of the peak response for each mouse against the value obtained for the internal standard experimental group (rAd_1). C. Hierarchical clustering (Euclidean/Ward.D2) performed on normalized T-cell response values defined as “Weak” (cluster 1) and “Strong” (clusters 2 and 3) vectors. Vectors in red were used to build the initial prediction model (see Model stability and confidence in the Results section).

Forty-one vectors classified in 13 categories of vaccines and all expressing the same antigen were evaluated and compared for their ability to induce an adaptive T-cell immune response after vaccination (S1 Table). The forty-one vectors included (i) recombinant viral vectors derived from adenovirus (rAd), vaccinia (VACC), modified vaccinia Ankara (MVA) and lentivirus (LV), (ii) recombinant bacteria vectors derived from Bacille de Calmette et Guérin (BCG), (iii) recombinant VLPs made of the AP205 [10] or Qbeta (Qb) [11] proteins from bacteriophage, the VP2 proteins from murine polyoma virus (MPY) [12] or murine pneumotropic virus (MPT), the Gag capsid proteins from murine leukaemia virus (MLV) [13], the core from hepatitis B virus (HBc), and (iv) plasmid encoding a recombinant protein (DNA) or recombinant MLV-VLPs (plasmoVLPs) [13,14]. Each vaccine platform was engineered to display or express the immunodominant LCMV gp33-41 epitope model antigen [15] in order to compare the different vaccine-induced CD8+ T-cell specific responses. In the framework of CompuVac (www.compuvac.eu), we standardized the method for measuring the gp33-41-specific T-cell response using tetramer staining (Fig 1A). Mice were immunized with each vector and we evaluated the gp33-41-specific T-cell response in PBMCs at days 5, 7 and 10, following the frequency of circulating gp33-41/H-2Db tetramer+ CD8+ T cells. In each experiment we included control mice that were injected with PBS or rAd (rAd_1 batch) to provide negative and positive controls. Data for each experimental group were normalized as the experimental to rAd vector response ratio allowing cross-laboratory data comparisons.

We observed a wide range of immune responses that were triggered by the different vectors. The maximal CD8+ T-cell expansion was induced with bacteriophage-derived VLPs, while very low but significant responses were observed with MPT and HBc VLPs (Fig 1B). Interestingly, different vector designs within the same vector platform led to different responses. As an example, Qb-derived VLPs induced variable CD8+ T-cell expansion depending on their production processes that were designed to modify their TLR-ligand composition (i.e. Qb_5 devoid of viral RNA and CpG in contrast to Qb_1; Fig 1A). We took into consideration all the vectors and performed hierarchical clustering on normalized values that defined 3 clusters (C). The first cluster comprised vectors with low ratio values, characterizing weak inducers of antigen-specific T cells, hereafter referred as “Weak” vectors. The other 2 clusters included vectors inducing high or intermediate responses, defining the “Strong” vector class. This class comprised the different recombinant viral vectors (rAd, MVA, VACC, LV) expressing rather than displaying the antigen, and which have been extensively developed as CD8+ T-cell vaccines [16–18]. It also contained bacteriophage-adjuvanted VLPs, in agreement with previous reports [10,19].

### Modelling strategy

As dendritic cell activation is key to the initiation of immune responses, we investigated whether transcriptome data from sorted spleen dendritic cells (DCs) sampled 6 hours after immunization could be predictive of the antigen-specific T-cell response measured several days later, at the peak of the response. To address this question, we devised a stepwise modelling scheme. DC-sorted transcriptome datasets were initially produced for 19 vectors on the Codelink platform, corresponding to 7 different vaccine platforms, for which the antigen-specific T-cell response was also measured (S1 Table).

The rationale for looking at signatures instead of individual genes was motivated by (i) the need to detect slight gene expression modifications (captured as the overall expression changes of correlated genes), (ii) the technical constraints of working on different microarray platforms (CodeLink, Illumina and Affymetrix), and (iii) the objective of producing a predictive model working across microarray platforms. Thus, our modelling scheme was based on our recently described strategy for signature discovery, using independent component analysis (ICA) followed by gene set enrichment analysis (GSEA) [20]. This allows circumventing the limitations due to the use of different platforms when analyzing individual gene expressions, by comparing statistical signature’s enrichment across datasets.

ICA is an unsupervised algorithm extracting independent components *Y* from original datasets *X* by searching for the demixing matrix *W*:

Y=X×W

*W* matrix is calculated by maximizing the non gaussianity of the components measured as the negentropy *J*:

$$J(y)=H(y_{Gauss})-H(y),$$

where *H*(*y*) and *H*(*y*_*Gauss*_) are the Shannon entropy for a vector *y* and a random Gaussian vector with same variance as *y* [21].

The use of ICA to analyze microarray data is justified by the hypothesis that *X* is a mix of signals from underlying cellular pathways. Therefore, columns of *Y* contain a summary of gene contributions in the extracted components. The RNA expression value of a gene is thus the superposition of several signals of this gene in each component which add up. From each component *y*, two reduced gene sets can be extracted by selecting genes with critical contribution on both sides of the distribution [22].

**Fig 2:**
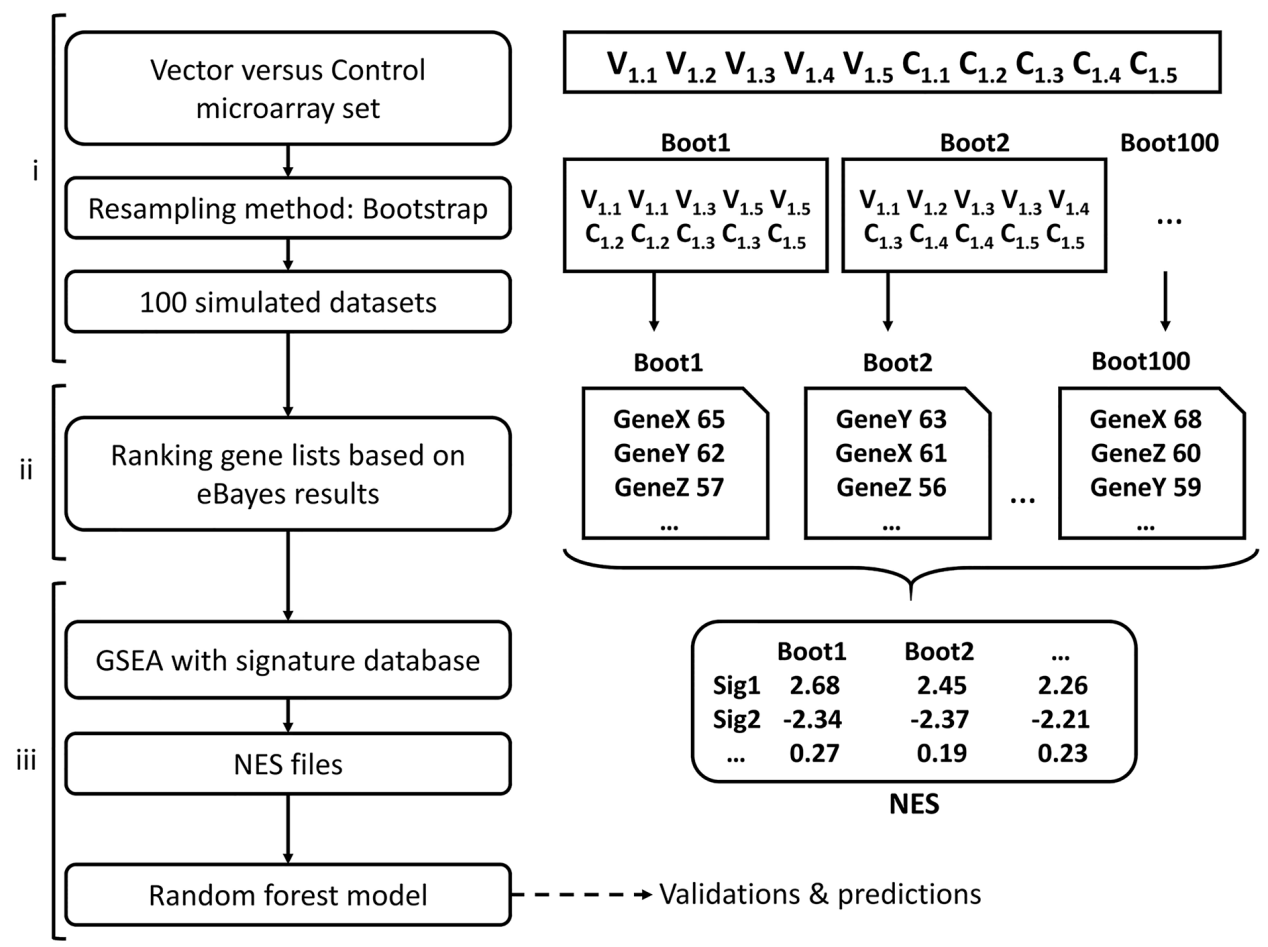
Modelling strategy. (i) For each pre-processed dataset, composed of microarray measures for mice injected with vector 1 (V1.1, V1.2 …) and control (C1.1, C1.2 …), one hundred datasets were created by bootstrapping samples among V and C. (ii) Ranked gene lists, according to the eBayes statistical comparison of vector and control conditions, were generated. (iii) Potential signatures were tested for enrichment on each of the 100 ranked gene lists by GSEA. The resulting NES matrix was then used to build the random forest model.

We first performed ICA on the 19 available datasets, yielding 210 molecular signatures characterizing the variability within each dataset, and likely linked to vector properties. We then analyzed the differential gene expression between the controls and the tested vectors using bootstrapping [23,24], in order to increase the model’s sensitivity. Bootstrapping consists in sampling series of additional datasets by randomly drawing samples with replacement of equal size from an original dataset, as described in Fig 2. We sampled 100 consecutive bootstrapped datasets from each of the 19 original datasets and generated 100 corresponding ranking lists of genes based on modified t-test statistics. The previously identified signatures were then tested for their behavior vis-à-vis the gene lists using GSEA, generating normalized enrichment scores (NES). Molecular signatures from GSEA software (>5000) were added at this step in order to increase the efficiency of the normalization procedure. NES of molecular signatures from ICA were then extracted for the next steps. This yielded a matrix, containing 1900 columns (100 bootstrapped datasets for each of the 19 original datasets) and 210 lines (the number of calculated NES). This matrix was then used to create random forest (RF) classification models (Fig 2). NES values and T-cell response classification were used as predictors and dependent variables, respectively, in the randomForest package, which as output provides classification results and associated probabilities for each T-cell response class.

### Model stability and confidence

An initial predictive model was built with 9 vector datasets (in red in Fig 1B) for which the antigen-specific T-cell responses were available (900 bootstrapped datasets and 100 signatures). Predictions of 10 additional datasets, including independent experiments done with the same or different batches of these vectors, were very consistent (see Tables 1 and S2). The model sensitivity for the “Weak” and “Strong” vector classes (respectively equal to the specificity for the “Strong” and “Weak” classes) are 0.89 and 0.98, respectively. The positive predictive value (PPV) is stable for the two classes (“Weak”: 0.96, “Strong”: 0.93). This 9-vector model is already efficient to classify the vector platform with 0.94 accuracy. These results led us to construct the final predictive model (called RFM model) including all the 19 datasets, based on the analysis of the 210 signatures across the 1900 bootstrapped datasets. This complete training set contained enough information to discriminate clearly between the 2 vector classes, as demonstrated by the misclassification rate parameter reaching zero after 100 simulated trees.

**Table 1:** Model’s sensitivity and accuracy.

Model	Sensitivity Strong (Specificity Weak)	Sensitivity Weak (Specificity Strong)	PPV Strong (NPV Weak)	PPV Weak (NPV Strong)	Accuracy
9-vector	0.98	0.89	0.93	0.96	0.94
RFM	**1**	**0.97**	**0.96**	**1**	**0.98**

**Fig 3:**
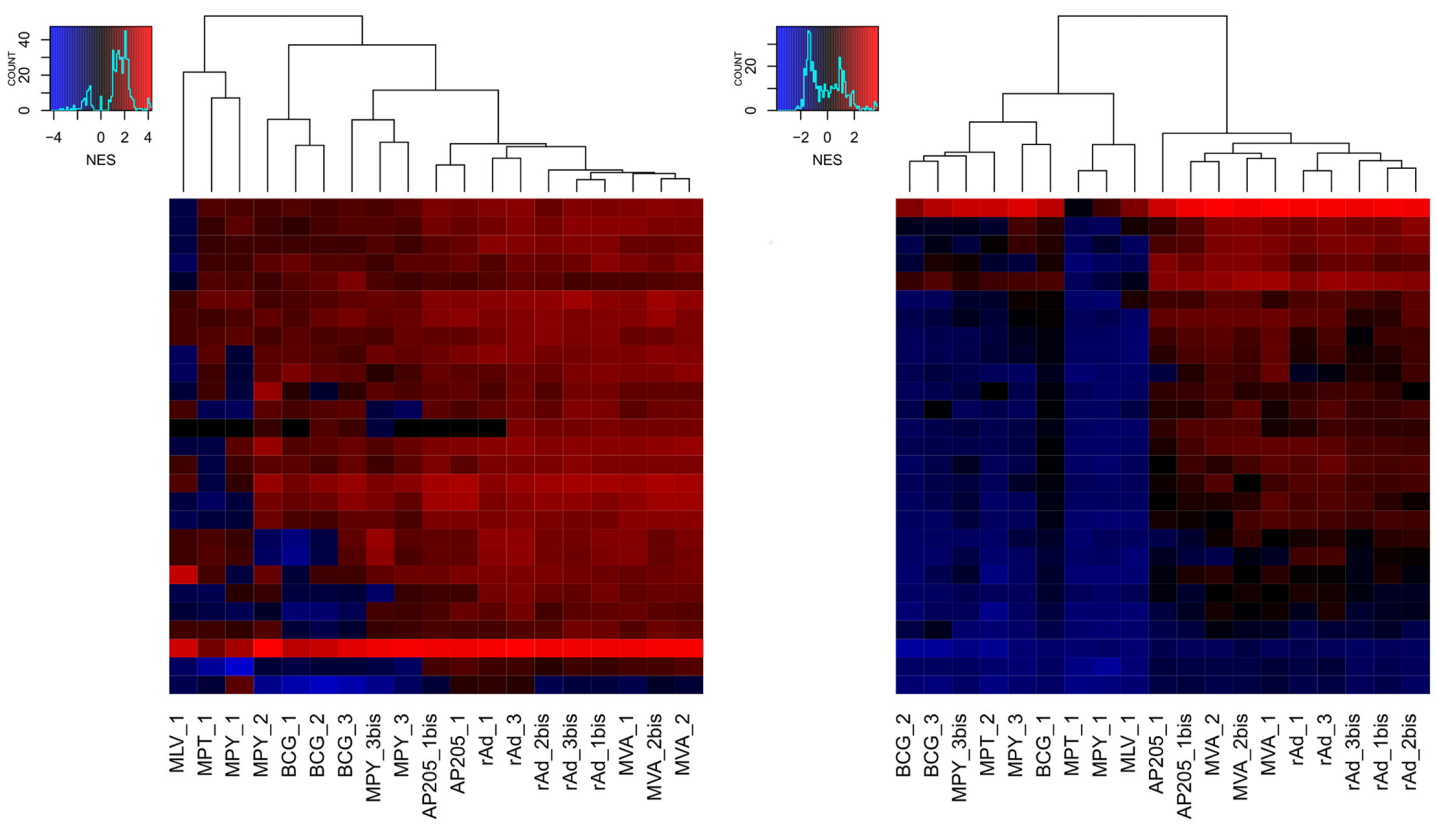
Hierarchical clustering (distance method: Euclidean; agglomeration method: Complete) of NES values of the 27 selected signatures, provided by the RFM model, on original vector datasets (A) and of mean NES values calculated on bootstrapped datasets (B).

The RandomForest algorithm provides a ranked list of the signatures based on their importance to the efficacy of the classification in the model. This score is based on the decrease of the Gini impurity criterion for each child node of a split. The result of this calculus is the mean of this decrease for each signature present in the trees of the forest. 27 most important signatures, having a mean decrease score higher than ten, were selected. Clustering methods were then applied (i) on NES values of these 27 signatures calculated on original datasets (Fig 3A) and (ii) on the mean NES values calculated on the bootstrapped datasets (Fig 3B). The interest of bootstrap is clearly revealed with clusters more explicitly defined after bootstrap.

We then asked whether RFM was biased toward particular vector datasets. We first used the leave-one-out methodology, where 19 models were iteratively built using only 18 out of 19 datasets, and then assessing how accurately such models predict the 100 bootstraps from the left-out dataset. All vectors were classified as expected for at least 96 of the 100 bootstrapped datasets, except MPY_3 for which 16 bootstrapped datasets were misclassified (S3 Table). This result shows overall very high prediction stability and no significant bias of the RFM model.

**Fig 4:**
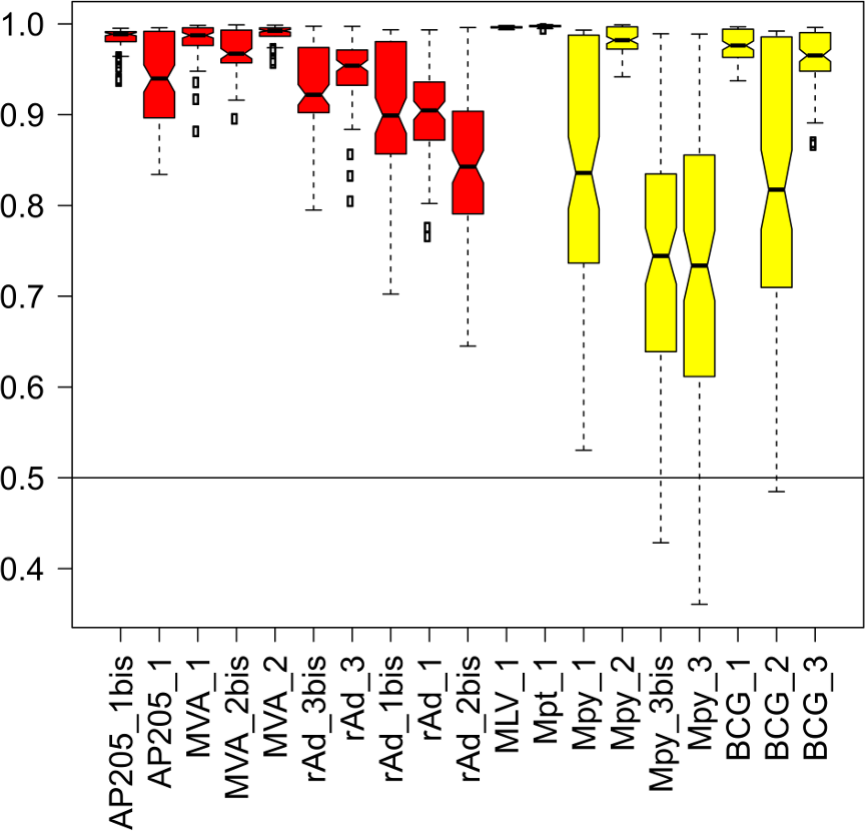
Vector prediction confidence. One hundred different models were created using one representative of each vector platform. The probabilities for a vector being classified as expected were calculated for its 100 bootstrapped datasets and averaged as a vector mean probability. Vector mean probabilities are displayed as boxplots. A value of 1 means that the bootstrapped dataset was successfully predicted 100 times over the 100 models. “Strong” and “Weak” vectors are colored red and yellow, respectively.

We verified that RFM was not biased for a given vector platform. One hundred new models were constructed, each based on one randomly selected representative of the 7 vector platforms (rAd, AP205, MVA, MPY, MPT, MLV and BCG). For each vector, the probabilities to be classified as expected were calculated and the prediction distribution across the 100 models is shown in Fig 4. Vaccines from the “Strong” vector class (in red) showed good consistency in their prediction distribution, with no value under 0.6 (100% confidence). Vaccines from the “Weak” vector class showed more variability: in particular, 2 MPY vaccines (MPY_3 & MPY_3bis; same vector batch (#3) used in 2 independent experiments) were not classified as expected in 16 models out of 100 (84% confidence); these 16 misclassifying models all used MPY_2 as the MPY representative. Note that this specific preparation (#2) of MPY vaccine was produced using baculovirus machinery in insect-derived cells, while the other MPYs were produced in yeast.

### Model validation with novel vectors

RFM was then used to predict the vector class of 4 new vectors belonging to 3 vector platforms: 2 batches of lentivirus (LV) vectors -a category of vaccine not represented during the model establishment, one new batch of AP205 (AP205_3) and one of MLV (MLV_2). We had independently determined that LV vectors induced strong antigen-specific T-cell responses after immunization and were classified in the “Strong” vector class (Fig 1). As shown in Tables 1 and 2A, these 4 bootstrapped datasets were classified as expected with high precision (>95%) while sensitivity and PPV of the model increased compared to the 9-vector model, especially the sensitivity for the “Weak” vector class now reaching 0.97 (from 0.89) with RFM. These results highlight that RFM (i) is not vaccine platform-dependent, (ii) correctly predicts a vector platform unknown to the model, and (iii) efficiently predicts both “Weak” and “Strong” vectors.

**Table 2:** Vector class prediction efficiency.

			RFM model predictions*	
	Vectors	Material	Strong	Weak
	LV_1	DC	**100**	0
	LV_2	DC	**100**	0
A	AP205_3	DC	3	**97**
	MLV_2	DC	5	**95**
	AP205_1	Spleen	**99**	1
	MVA_1	Spleen	**100**	0
B	rAd_1	Spleen	**98**	2
	MLV_1	Spleen	9	**91**
	MPT_1	Spleen	0	**100**
	AP205_1	PBMC	**73**	27
	MVA_1	PBMC	**90**	10
	Qb_1	PBMC	**99**	1
	Qb_2	PBMC	**95**	5
C	Qb_3	PBMC	**100**	0
	Qb_4	PBMC	**100**	0
	Qb_5	PBMC	**98**	2
	MLV_1	PBMC	0	**100**
	MPT_1	PBMC	0	**100**
	rAd_1_6	Spleen	**98**	2
D	rAd_1_48	Spleen	0	**100**
	rAd_1_72	Spleen	2	**98**

*Number of the 100 bootstrapped datasets predicted as “Strong” or “Weak”.

### Model prediction of whole spleen and PBMC data

RFM was built on transcriptome data obtained from sorted spleen DCs. In our next experiment, we assessed whether RFM would be sensitive enough to classify transcriptome datasets derived from whole spleen samples obtained 6 hours after immunization, where DCs represent 1–2% of total splenocytes. As summarized in Table 2B, all bootstrapped datasets from whole spleens were well classified, with at least 91% of the expected classification, thus demonstrating our model’s sensitivity in classifying vectors in whole spleen transcriptome datasets.

We then tested microarray datasets for whole spleen samples obtained 6, 48 and 72 hours after vaccination with one vector, the rAd vector that we used as a standard. Strikingly, only datasets sampled 6 hours after injection were classified as expected (as “Strong”) (Table 2D).

Similarly, we tested the performance of our model in classifying vectors using PBMC-derived microarray datasets. The rationale for this experiment is that PBMCs, less than 1% of which are DCs, offer a more accessible sample source than spleen, especially in humans. As shown in Table 2C, all but one vectors were classified as expected with high precision (≥ 90%). AP205_1 was classified as expected, though with less confidence (73%).

### Model prediction of human PBMC data

Finally, we tested whether our model could classify datasets obtained from the literature. We found datasets from the Merck Ad5/HIV trial reported by Zak et al. [25] PBMC transcriptome data were generated from samples obtained at 6, 24 and 72 hours after vaccination. We bootstrapped the samples of Zak et al., taking patient-paired samples before and after vaccination. 100% and 91% of the bootstrapped paired samples were predicted as “Strong” at 24 and 72 hours, respectively (Table 3), in line with the authors’ original observations. The same analysis performed with the 6-hour time point gave a “Strong” prediction for 31% of the bootstrapped paired samples. The latter finding is consistent with the conclusion of Zak et al. that transcriptomic modifications at 6 hours were not significant. These results demonstrate the capacity of RFM generated from mouse DC transcriptome datasets to classify human PBMC datasets.

**Table 3:** Predictions of human PBMC transcriptome data derived 6, 24 and 72 hours after vaccination by MRKAd5/HIV published in [25].

		RFM model predictions*	
Time point	Material	Strong	Weak
6 h	PBMCs	31	**69**
24 h	PBMCs	**100**	**0**
72 h	PBMCs	**91**	**9**

*Number of the 100 bootstrapped datasets predicted as “Strong” or “Weak”.

### Biological insight

**Fig 5:**
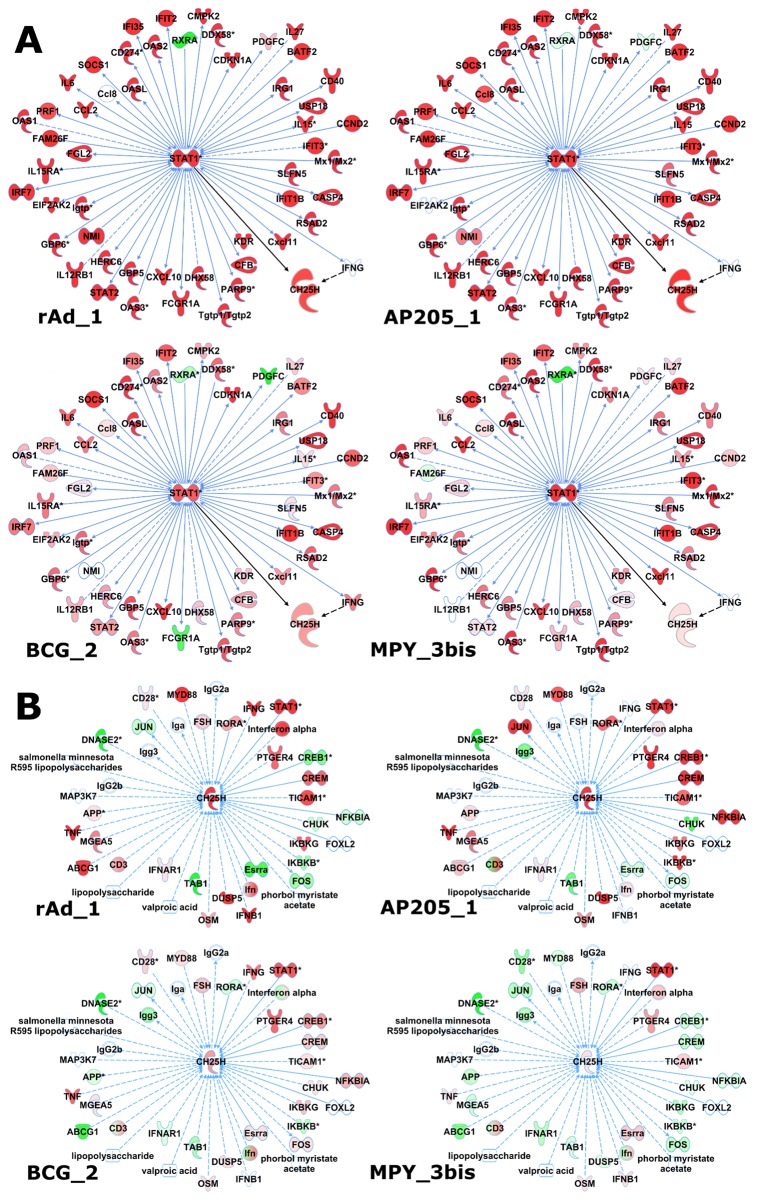
Gene network analysis in “Weak” and “Strong” vectors. A: Selection of STAT-1 related genes derived from Sig1 of the RFM model were targeted on Ingenuity Pathway Analysis (IPA). B: CH25H, a gene selected in one of the other 26 important signatures of the model, was targeted as the key gene on IPA. The grow functionality was used to display all known direct and indirect interactions with CH25H, except miRNA. The biological interactions of CH25H are displayed on A (black arrows). Colors depend on statistical analyses (red: upregulated, green: downregulated) performed on rAd_1, AP205_1, MPY_3bis and BCG_2 vector datasets; color intensities were set to be in the same range in all experiments.

Biological annotation of the 27 most important signatures of RFM reveals one signature (Sig1) with statistical functional enrichments related to immune processes (FDR p-values 10^−4^–10^−8^). This signature is highly focused on STAT-1 with 51 genes having strong biological connections (Fig 5A). Interestingly, Sig1 is upregulated in all the vectors, but with higher intensity in the “Strong” as compared to the “Weak” vectors.

No specific molecular pathway was clearly identified by QIAGEN’s Ingenuity Pathway Analysis (IPA) functional analysis for the other 26 important signatures in our model. However, visual inspection of these signatures identified the CH25H gene as highly modulated by strong vectors. Since this gene has been recently described as playing a role in DC maturation [26], we analyzed its network of connected genes with IPA (Fig 5B). This network was also globally more modulated by “Strong” rather than “Weak” vectors, and comprised genes implicated in DC function such as MYD88, DUSP5 and ABCG1.

## Discussion

Understanding and predicting innate immune response to vector platforms is primordial for fast and effective production of new vaccination or gene therapy protocols. Systems biology tools efficiently extract information from large datasets in computing predictive models and have already played a major role in recent discoveries in this field [5,27]. In this paper, we initially focused on early transcriptomic changes of DCs since these are first-line players in the innate immune response and directly contribute to the triggering of the adaptive response. Our aim was to identify transcriptomic signatures predictive of the late CD8+ CTL responses to the LCMV gp33-41 model antigen conveyed by a variety of vaccine vectors.

Based on molecular signatures extracted using the non-supervised ICA method [20,22], we produced and validated a prediction model taking into account 19 available datasets generated with different vector platforms. We chose the random forest learning algorithm for its reported efficiency among classification methodologies [28–30]. The originality of our strategy was the use of signatures rather than genes to classify samples. Our results showed that this model consistently predicts both “Weak” and “Strong” vectors, with greater confidence for the latter. This suggests that there are shared gene expression modifications induced by “Strong” vectors, while changes induced by “Weak” vectors are more diverse. Consistent with this, Li et al. recently reported that different types of vaccine lead to different transcriptomic modifications in humans 3 days after vaccination [31], with vaccines inducing high transcriptomic modifications being those that induce robust antibody responses.

Among the 27 signatures selected for their importance in the RFM model, one (Sig1; see S4 Table) is related to immune components, including “viral infection”, “role of RIG1-like receptors in antiviral innate immunity” and “interferon signalling” pathways. Previous studies have characterized gene expression modifications in the early stages of vaccination consistent with Sig1 annotation. Querec et al. investigated the transcriptome of patient PBMCs at days 0, 1, 3, 7 and 10 after vaccination with yellow fever vaccine [9]. Of 65 regulated genes, 26 were related in part to interferon and the antiviral response, including MX1, IFIT1, IFIT2, IFIT3, OAS1, OAS2, OAS3 and OASL, and 7 were related to signal transduction, including STAT1 and IRF7. Similarly, Zak et al. [25] applied the modular transcriptome analysis framework described in Chaussabel et al. [32] to study the innate immune response to MRKAd5/HIV in PBMCs 6, 24, 72 and 168 hours after patient vaccination. They identified genes highly regulated at 6 and 24 hours, including STAT1, STAT2, IFITs, MXs and OASs (also identified in Querec et al.). Strikingly, all these genes are also part of Sig1, emphasizing further their key role in the early response to the vaccine. Furthermore, DDX60, a newly described antiviral factor that induces Rig-1-like receptor-mediated signaling [33], present in Sig1, was reported by Querec et al. as well [9]. Interestingly, Sig1 is upregulated in vaccinated samples compared to control group, but to a lesser extent in “Weak” vs. “Strong” vectors (see Figs 3 and 5).

Our cross-analysis of Zak et al.’s microarray data on Merck Ad5/HIV-vaccinated human PBMC samples, which yield good predictions for the 24- and 72-hour time points, demonstrates that our prediction model, solely based on mouse DC-sorted transcriptome data, efficiently predicts human transcriptome data. This can be explained by the high similarity of gene expression in immunological cell lineages between mice and humans [34], although the kinetics of the immune response to vaccine is different.

No specific molecular pathway was clearly identified by IPA annotations for the other 26 important signatures in our model. This is somewhat surprising since these signatures have been selected by the model to best distinguish “Strong” and “Weak” vectors and are therefore expected to represent differentially regulated biological pathways. In this line, none of the 27 signatures corresponds to a peculiar behavior of a vector but they rather reveal similar behavior within “Strong” or “Weak” groups (Fig 3). Moreover, the identified signatures were extracted from 13 out of 19 different vector datasets (9 “Strong” and 4 “Weak” vectors). We believe that these signatures are unlikely artifactual but related to yet undefined biological processes. Indeed, the constant improvement of annotation databases can reveal secondary or additional functions of genes. For example, CH25H, a gene found in one of the 26 signatures and clearly upregulated in “Strong” vectors, is primarily involved in cholesterol metabolism, but has recently been shown to play a role in the early stage of DC maturation [26]. Fig 5 shows how the expression of this gene is related to dendritic cell through direct or indirect interactions with STAT-1 or IFNγ, both members of Sig1, and with several genes known to be important in early dendritic cell activation: for example, MYD88 is a gene involved in toll-like receptor signaling [35], DUSP5 is known to be upregulated during dendritic cell maturation [36], and ABCG1 is a gene playing a role in adaptive immune responses [37]. The comparative analysis of gene expression modulation of this interaction network shown in Fig 5 reveals a similar pattern of differential expression for “Strong” vectors (rAd_1, AP205_1) different than that observed for “Weak” vector datasets (BCG_2, MPY_3bis). This again points at a significant difference in early dendritic cell activation-related gene behavior in “Strong” vs. “Weak” vectors.

**Fig 6:**
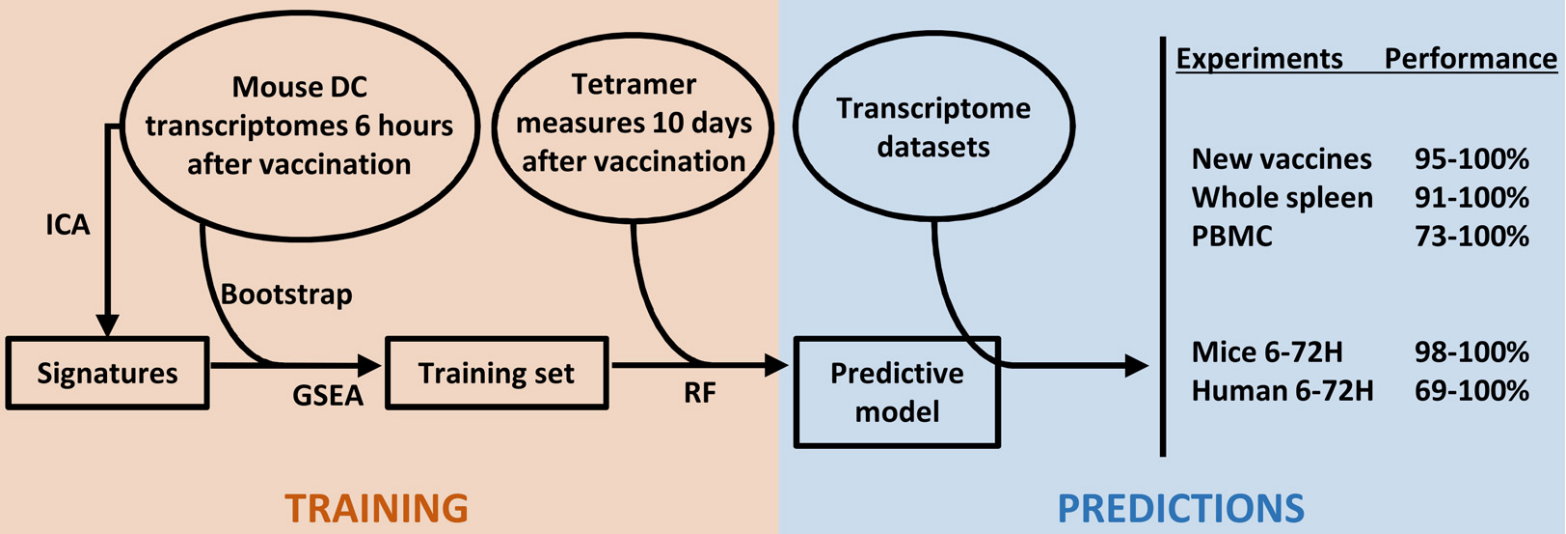
Strategy followed in this study for a high-performance predictive model of transcriptome datasets obtained from new vector platforms, cells from whole organs (spleen, blood). RF: random forest.

Altogether, our results underline the relevance of the CompuVac initiative that consisted in producing, in a standardized manner, immunological and transcriptome data related to vaccine candidates in order to predict their capacity to elicit strong antigen-specific responses. Our model was based on transcriptome data from sorted spleen DCs of mice vaccinated with various “Strong” and “Weak” T-cell inducer vectors. This prediction model accurately predicted the behavior of these and other candidate vaccines only 6 hours after injection. The model was powerful enough to produce a relevant vector classification even when using whole mouse spleen and PBMCs, or even human PBMCs (Fig 6), and across 3 microarray platforms (CodeLink, Illumina and Affymetrix). The accuracy and sensitivity of the model are likely high because it is built with very different vaccine platforms therefore representative of possible vector behaviors in triggering the early immune response. This study further supports the potential of systems immunology approaches in facilitating the development and characterization of vaccines, offering robust *in silico* solutions to study the early events of the immune response to vaccines.

## Material and Methods

### Ethics statement

Experimental protocols complied with French law (Décret: 2001–464 29/05/01) and EEC regulations (86/609/CEE) for the care and use of laboratory animals and were carried out under Authorization for Experimentation on Laboratory Animals Number 75-673-R. Our animal protocol (Ce5/2009/042) was approved by the “Charles Darwin” Ethics Committee for Animal Experimentation (CNREEA 05) and performed in the licensed animal facility A75-13-08.

### Vector platforms

Recombinant adenovirus- and MVA-derived viral vectors, BCG-derived bacterial vector, AP205 [10] or Qb [11] bacteriophage-, MPT- and MPY- [12] or MLV-derived [13] VLPs used as an antigenic platform and DNA vaccines were included in this study. According to the CompuVac evaluation scheme, each vaccine platform was engineered to display / express the LCMV gp33-41 model antigen [15] in order to measure the vaccine-induced T-cell specific responses and dendritic cell transcriptome changes (see following sections). The sequence IITSIKAVYNFATCGILAL corresponding to the GP33-41 epitope flanked upstream and downstream by 5 of its natively neighboring amino acids was used. The 53 vectors considered in this paper (S1 Table) are displayed in 13 vector platforms 7 of which were used for a training set (rAd, MVA, AP205, MPT, MPY, MLV and BCG) and 2 for prediction of new platforms (LV and Qb).

### Evaluation of antigen-specific T-cell responses

Groups of three to five 7-week-old female C57BL/6 mice (Charles River, France and Germany) were immunized with a controlled quantity of vector particles as defined in CompuVac assay protocols (www.compuvac.eu). For monitoring T-cell responses, each vector was injected with its “best” route of administration: subcutaneously for VLP vectors; intramuscularly for recombinant antigen-expressing vectors and by intra-dermally by gene gun for DNA vaccines. Control mice were injected with 100 μL of phosphate buffered saline solution (PBS). For each vector (n = 41), the T-cell immune response measurement was performed independently one to three times. T-cell immune responses induced against the LCMV gp33-41 model antigen were measured by MHC-I gp33-41/H-2Db tetramer (ProImmune, UK) staining of PBMCs at 5, 7 and 10 days after injection. The highest measure was kept for each mouse and the mean value was then calculated for the group. Values were normalized against measures monitored in parallel in mice immunized with the rAd_1 control vector.

### Microarray data

Experimental groups comprised of 3 to 6 mice immunized with vaccine candidates by the intravenous route. Mice were sacrificed 6 hours after immunization. Spleen DCs were purified with CD11c+-conjugated MACS magnetic beads (Miltenyi Biotec) according to the manufacturer's instructions. After incubation for 20 minutes at 4°C, cells were washed and passed over a MACS column. Purity was checked routinely by FACS and found to be greater than 96±2%. 2x10^6^ CD11c+ cells were used for total RNA extraction using Nucleospin RNAII (Macherey Nagel). For test dataset generation, whole PBMCs and/or whole splenocytes and/or sorted spleen DCs were collected at 6 hours, and at 48- and 72-hour time points for the kinetic follow-up. RNA was checked for quality using gel electrophoresis and for quantity using a Nanodrop spectrophotometer (Thermo Scientific). Microarrays were performed using either Applied Microarrays (CodeLink Mouse Whole Genome Bioarray) or Illumina (WG6 Mouse BeadArray) technologies (S1 Table). The MessageAmp II aRNA Amplification Kit (Ambion) was used for cDNA and cRNA production from 1 μg of total RNA. 10 μg of amplified cRNA was subsequently fragmented and hybridized for 20 hours using the Applied Microarrays hybridization and washing buffer kit. Slides were scanned using the GenePix Personal 4100A scanner for CodeLink array or the Illumina BeadArray 500GX Reader for Illumina array. Hybridization and raw data extraction were performed using either GenePix Pro 6.0 (for CodeLink array) or BeadStudio (for Illumina array) software, respectively (GEO accession GSE66991).

Each tested vector dataset comprised “vector-immunized” and corresponding PBS control samples. Quantile normalization was performed with the limma package [38] on R software [39], and then a log2 transformation was applied. Probes with a detection p-value above 0.05 in all samples in a dataset were discarded.

### Signature database and enrichment analysis

Following our two-step ICA→GSEA signature discovery strategy [20], signatures were extracted using the fastICA algorithm R package [40] following modifications in [22]. Parameters were set as default, except for the unmixing matrix A^-1^ convergence threshold set to 10^-6^. Ranked gene lists were calculated using the limma modified t-test. ES were calculated using GSEA [41] with the pre-ranked gene list protocol. Normalized ES are then calculated based on the permutation performed on gene sets collection, allowing comparison between experiments. The ICA-extracted signature database was complemented with the MsigDB C2 (curated gene sets of biological pathways) and C5 (Gene Ontology gene sets) databases (www.broad.mit.edu/gsea) in order to increase universe of genes available for permutation of gene sets. Signatures with fewer than 7 detected genes were ignored.

### Random forest classification and validation

For each model produced in the Results section, classification was performed on a matrix of fastICA extracted signature NES values (see above section) calculated for bootstrapped vector datasets (100 bootstraps per vector dataset), using the random forest algorithm implemented in the randomForest R package to produce a forest of 2000 trees [42]. The number of randomly selected signatures used at each of the 2000 runs was set according to the *mtry* function implemented in the randomForest package. The class prediction of the new dataset was deduced by the probability to be “Weak” or “Strong” > 0.5. The overall vector class was then obtained as the majority of “Weak” or “Strong” class assignments over the 100 bootstraps.

For classification model validation, we implemented the leave-one-out methodology consisting in creating models with n-1 datasets, where n is the total number of datasets, and classifying the dataset left out. In addition, we implemented a “multi-model” methodology based on the classification of bootstrapped datasets over 100 models created as above. Each model was computed on an NES matrix of a random selection of one representative vector dataset of each of the 7 represented vector platforms (see Vector platforms section and S1 Table). Vector mean probabilities were calculated as the average probability of being “Weak” or “Strong” over the 100 bootstrapped vector datasets, and their distribution over the 100 models was analyzed.

### Signature annotation

For biological insight evaluation of the signatures, microarray data were analyzed through the use of QIAGEN’s Ingenuity Pathway Analysis (IPA, QIAGEN Redwood City, www.qiagen.com/ingenuity).

## Supporting Information

S1 Table List of studied vectors. (PDF)

S2 Table Classification of vectors based on the 9-vector model. (PDF)

S3 Table Predictions of dendritic cell transcriptome data derived from mice vaccinated with different vectors in the leave-one-out validation step. (PDF)

S4 Table List of genes in Sig1. (PDF)
