# Supplementary material for: Early Transcriptome Signatures from Immunized Mouse Dendritic Cells Predict Late Vaccine-Induced T-Cell Responses
Source: PLoS Comput Biol. 2016 Mar 21;12(3):e1004801. doi: 10.1371/journal.pcbi.1004801 (PMC4801398; doi:10.1371/journal.pcbi.1004801)
Supplement: S1 Table — (PDF) [file pcbi.1004801.s001.pdf]

S1 Table: List of studied vectors.

Vector ID (a)	Vector platform	gp33/41 T-cell response (b)	Microarray (c)
ADN_1	DNA	Yes	
ADN_2	DNA	Yes	
ADN_3	DNA	Yes	
ADN_4	DNA	Yes	
AP205_1	VLP bacteriophage	Yes	DC/SPL*/PBMC*
AP205_1bis	VLP bacteriophage	Yes	DC
AP205_3	VLP bacteriophage		DC
AP205_4	VLP bacteriophage	Yes	
AP205_5	VLP bacteriophage	Yes	
AP205_6	VLP bacteriophage	Yes	
AP205_7	VLP bacteriophage	Yes	
BCG_1	BCG-derived bacterial vector	Yes	DC
BCG_2	BCG-derived bacterial vector		DC
BCG_3	BCG-derived bacterial vector		DC
BCG_4	BCG-derived bacterial vector	Yes	
HBC_1		Yes	
HBC_2		Yes	
HBC_3		Yes	
MLV_1	VLP Moloney murine leukaemia retrovirus	Yes	DC/SPL*/PBMC*
MLV_2	VLP Moloney murine leukaemia retrovirus		DC
MPT_1	VLP murine pneumotropic virus	Yes	DC/SPL*/PBMC*
MPT_2	VLP murine pneumotropic virus	Yes	
MPT_3	VLP murine pneumotropic virus	Yes	
MPY_1	VLP murine polyomavirus	Yes	DC
MPY_2	VLP murine polyomavirus	Yes	DC
MPY_3	VLP murine polyomavirus		DC
MPY_3bis	VLP murine polyomavirus		DC
MVA_1	Modified vaccinia Ankara	Yes	DC/SPL*/PBMC*
MVA_2	Modified vaccinia Ankara		DC
MVA_2bis	Modified vaccinia Ankara		DC
MVA_3	Modified vaccinia Ankara	Yes	
LV_1	Lentivirus	Yes	DC*
LV_2	Lentivirus	Yes	DC*
Plasmo_1		Yes	
Plasmo_2		Yes	
Plasmo_3		Yes	
Plasmo_4		Yes	
Plasmo_6		Yes	
QB_1	Qb bacteriophage	Yes	PBMC*
QB_2	Qb bacteriophage	Yes	PBMC*
QB_3	Qb bacteriophage	Yes	PBMC*
QB_4	Qb bacteriophage	Yes	PBMC*
QB_5	Qb bacteriophage	Yes	PBMC*
QB_6	Qb bacteriophage	Yes	
rAd_1	Adenovirus	Yes	DC/SPL*
rAd_1bis	Adenovirus		DC
rAd_2bis	Adenovirus		DC
rAd_3	Adenovirus		DC
rAd_3bis	Adenovirus		DC
rAd_4	Adenovirus	Yes	
rAd_5	Adenovirus	Yes	
VACC_1	Vaccinia	Yes	
VACC_2	Vaccinia	Yes	

(a) Numbers refer to independent vector preparations; "bis" corresponds to a duplicate analysis of the same corresponding batch.

(b) This column indicates the 41 vectors for which the gp33-41 specific T-cell response was assessed.

(c) This column indicates vectors for which sorted spleen dendritic cells (DC), whole spleen (SPL) and/or PBMC microarray data are available. Microarray datasets were produced with CodeLink except those marked by an asterisk produced with Illumina. Except for the AP205_3, MLV_2, LV_1 and LV_2 datasets, which were generated for validation purposes, the other 19 DC datasets were used in the training set.
