# Supplementary material for: Early Transcriptome Signatures from Immunized Mouse Dendritic Cells Predict Late Vaccine-Induced T-Cell Responses
Source: PLoS Comput Biol. 2016 Mar 21;12(3):e1004801. doi: 10.1371/journal.pcbi.1004801 (PMC4801398; doi:10.1371/journal.pcbi.1004801)
Supplement: S2 Table — (PDF) [file pcbi.1004801.s002.pdf]

S2 Table: Classification of vectors based on the 9-vector model.

		# predicted datasets	
Vectors	Expected*	Strong	Weak
MVA_2	Strong	**100**	0
MVA_2bis	Strong	**100**	0
rAd_1bis	Strong	**95**	5
rAd_2bis	Strong	**94**	6
rAd_3bis	Strong	**98**	2
rAd_3	Strong	**100**	0
MPY_3	Weak	8	**92**
MPY_3bis	Weak	16	**84**
BCG_2	Weak	0	**100**
BCG_3	Weak	20	**80**

*Strong and weak class attribution is based on tetramer complex measures
