# Supplementary material for: Early Transcriptome Signatures from Immunized Mouse Dendritic Cells Predict Late Vaccine-Induced T-Cell Responses
Source: PLoS Comput Biol. 2016 Mar 21;12(3):e1004801. doi: 10.1371/journal.pcbi.1004801 (PMC4801398; doi:10.1371/journal.pcbi.1004801)
Supplement: S3 Table — (PDF) [file pcbi.1004801.s003.pdf]

S3 Table: Predictions of dendritic cell transcriptome data derived from mice vaccinated with different vectors in the leave-one-out validation step.

		# predicted datasets	
Vectors	Expected*	Strong	Weak
AP205_1	Strong	**96**	4
AP205_1bis	Strong	**100**	0
MVA_2	Strong	**100**	0
MVA_2bis	Strong	**100**	0
MVA_1	Strong	**100**	0
rAd_1	Strong	**98**	2
rAd_1bis	Strong	**99**	1
rAd_2bis	Strong	**96**	4
rAd_3bis	Strong	**100**	0
rAd_3	Strong	**100**	0
MLV_1	Weak	0	**100**
MPT_1	Weak	0	**100**
MPY_2	Weak	0	**100**
MPY_1	Weak	0	**100**
MPY_3	Weak	16	**84**
MPY_3bis	Weak	1	**99**
BCG_1	Weak	0	**100**
BCG_2	Weak	0	**100**
BCG_3	Weak	0	**100**

*Strong and weak classes are based on tetramer complex measures
