# Supplementary material for: Early Transcriptome Signatures from Immunized Mouse Dendritic Cells Predict Late Vaccine-Induced T-Cell Responses
Source: PLoS Comput Biol. 2016 Mar 21;12(3):e1004801. doi: 10.1371/journal.pcbi.1004801 (PMC4801398; doi:10.1371/journal.pcbi.1004801)
Supplement: S4 Table — (PDF) [file pcbi.1004801.s004.pdf]

S4 Table: List of genes in Sig1.

Symbol	Entry Gene Name
2610524H06Rik	RIKEN cDNA 2610524H06 gene
2810405F15Rik	RIKEN cDNA 2810405F15 gene
4930599N23Rik	RIKEN cDNA 4930599N23 gene
9430034N14Rik	RIKEN cDNA 9430034N14 gene
9830107B12Rik (includes others)	RIKEN cDNA B430306N03 gene
ABTB2	ankyrin repeat and BTB (POZ) domain containing 2
Ahsa2	AHA1, activator of heat shock protein ATPase 2
AIDA	axin interactor, dorsalization associated
AIM2	absent in melanoma 2
ANXA4	annexin A4
APOD	apolipoprotein D
Apol9a/Apol9b	apolipoprotein L 9b
ARHGAP8/PRR5-ARHGAP8	Rho GTPase activating protein 8
ARSI	arylsulfatase family, member I
ART3	ADP-ribosyltransferase 3
ASB13	ankyrin repeat and SOCS box containing 13
ATP10A	ATPase, class V, type 10A
BATF2	basic leucine zipper transcription factor, ATF-like 2
BBX	bobby sox homolog (Drosophila)
BST2	bone marrow stromal cell antigen 2
BTBD16	BTB (POZ) domain containing 16
BZRAP1	benzodiazapine receptor (peripheral) associated protein 1
C11orf85	chromosome 11 open reading frame 85
C130026I21Rik (includes others)	RIKEN cDNA C130026I21 gene
C15orf48	chromosome 15 open reading frame 48
C19orf12	chromosome 19 open reading frame 12
C1orf192	chromosome 1 open reading frame 192
C3AR1	complement component 3a receptor 1
C4orf32	chromosome 4 open reading frame 32
CA13	carbonic anhydrase XIII
CASP4	caspase 4, apoptosis-related cysteine peptidase
CCL2	chemokine (C-C motif) ligand 2
CCL7	chemokine (C-C motif) ligand 7
Ccl8	chemokine (C-C motif) ligand 8
CCL13	chemokine (C-C motif) ligand 13
CCND2	cyclin D2
CD40	CD40 molecule, TNF receptor superfamily member 5
CD274	CD274 molecule
CDC42BPG	CDC42 binding protein kinase gamma (DMPK-like)
CDH15	cadherin 15, type 1, M-cadherin (myotubule)
CDH22	cadherin 22, type 2
CDKN1A	cyclin-dependent kinase inhibitor 1A (p21, Cip1)
CFB	complement factor B
CFLAR	CASP8 and FADD-like apoptosis regulator
Clec2e	C-type lectin domain family 2, member e
CMPK2	cytidine monophosphate (UMP-CMP) kinase 2, mitochondrial
COMP	cartilage oligomeric matrix protein
Creb5	cAMP responsive element binding protein 5
CWH43	cell wall biogenesis 43 C-terminal homolog (S. cerevisiae)
CXCL9	chemokine (C-X-C motif) ligand 9
CXCL10	chemokine (C-X-C motif) ligand 10
Cxcl11	chemokine (C-X-C motif) ligand 11
DAXX	death-domain associated protein
DDX4	DEAD (Asp-Glu-Ala-Asp) box polypeptide 4
DDX58	DEAD (Asp-Glu-Ala-Asp) box polypeptide 58
DDX60	DEAD (Asp-Glu-Ala-Asp) box polypeptide 60

DHX58	DEXH (Asp-Glu-X-His) box polypeptide 58
DNAJA4	DnaJ (Hsp40) homolog, subfamily A, member 4
DOPEY2	dopey family member 2
EIF2AK2	eukaryotic translation initiation factor 2-alpha kinase 2
ELL3	elongation factor RNA polymerase II-like 3
EN2	engrailed homeobox 2
EPS8L2	EPS8-like 2
FAM162B	family with sequence similarity 162, member B
FAM26F	family with sequence similarity 26, member F
Fbxw17	F-box and WD-40 domain protein 17
FCGR1A	Fc fragment of IgG, high affinity Ia, receptor (CD64)
FCGR3A	Fc fragment of IgG, low affinity IIIa, receptor (CD16a)
FGL2	fibrinogen-like 2
FNDC3A	fibronectin type III domain containing 3A
FOXF1	forkhead box F1
FPR2	formyl peptide receptor 2
Gbp2	guanylate binding protein 2
GBP4	guanylate binding protein 4
GBP5	guanylate binding protein 5
GBP7	guanylate binding protein 7
Gbp6 (includes others)	guanylate binding protein 6
GCA	grancalcin, EF-hand calcium binding protein
GCH1	GTP cyclohydrolase 1
GJD3	gap junction protein, delta 3, 31.9kDa
GLIPR2	GLI pathogenesis-related 2
Gm4951	predicted gene 4951
Gm4955	predicted gene 4955
Gm8995	predicted gene 8995
Gm20559	predicted gene, 20559
Gm8672 (includes others)	predicted gene 906
GNB4	guanine nucleotide binding protein (G protein), beta
Gpr33	G protein-coupled receptor 33
GPSM2	G-protein signalling modulator 2
Gvin1 (includes others)	GTPase, very large interferon inducible 1
GZMB	granzyme B (granzyme 2, cytotoxic T-lymphocyte-associated
H2-T24	histocompatibility 2, T region locus 24
HAPLN1	hyaluronan and proteoglycan link protein 1
HDC	histidine decarboxylase
HELZ2	helicase with zinc finger 2, transcriptional coactivator
HERC6	HECT and RLD domain containing E3 ubiquitin protein ligase
IFI35	interferon-induced protein 35
IFI44	interferon-induced protein 44
Ifi204 (includes others)	interferon activated gene 204
IFIH1	interferon induced with helicase C domain 1
IFIT2	interferon-induced protein with tetratricopeptide repeats 2
IFIT3	interferon-induced protein with tetratricopeptide repeats 3
IFIT1B	interferon-induced protein with tetratricopeptide repeats 1B
IFITM2	interferon induced transmembrane protein 2
IFNA1/IFNA13	interferon, alpha 1
IFNG	interferon, gamma
IGF2BP3	insulin-like growth factor 2 mRNA binding protein 3
Igtp	interferon gamma induced GTPase
Iigp1	interferon inducible GTPase 1
IL6	interleukin 6 (interferon, beta 2)
IL15	interleukin 15
IL27	interleukin 27
IL12RB1	interleukin 12 receptor, beta 1
IL15RA	interleukin 15 receptor, alpha
IL17RE	interleukin 17 receptor E
IL1RN	interleukin 1 receptor antagonist
ILDR1	immunoglobulin-like domain containing receptor 1

INSL6	insulin-like 6
INTS7	integrator complex subunit 7
IRF7	interferon regulatory factor 7
Irg1	immunoresponsive gene 1
IRGM	immunity-related GTPase family, M
ISG20	interferon stimulated exonuclease gene 20kDa
JHDM1D	jumonji C domain containing histone demethylase 1 homolog D
KAT6B	K(lysine) acetyltransferase 6B
KDR	kinase insert domain receptor (a type III receptor tyrosine
KLHL3	kelch-like family member 3
LAP3	leucine aminopeptidase 3
LGALS9	lectin, galactoside-binding, soluble, 9
LHX2	LIM homeobox 2
LHX4	LIM homeobox 4
LIMA1	LIM domain and actin binding 1
LSM14A	LSM14A, SCD6 homolog A (S. cerevisiae)
MARCH5	membrane-associated ring finger (C3HC4) 5
MARCKSL1	MARCKS-like 1
MAX	MYC associated factor X
MED13L	mediator complex subunit 13-like
MID1	midline 1 (Opitz/BBB syndrome)
MITD1	MIT, microtubule interacting and transport, domain containing
MLKL	mixed lineage kinase domain-like
MMP13	matrix metallopeptidase 13 (collagenase 3)
MOCOS	molybdenum cofactor sulphurase
MOV10	Mov10, Moloney leukaemia virus 10, homolog (mouse)
Ms4a4b (includes others)	membrane-spanning 4-domains, subfamily A, member 4B
MS4A6A	membrane-spanning 4-domains, subfamily A, member 6A
Ms4a6b	membrane-spanning 4-domains, subfamily A, member 6B
MTHFR	methylenetetrahydrofolate reductase (NAD(P)H)
Mx1/Mx2	myxovirus (influenza virus) resistance 1
MXD1	MAX dimerization protein 1
MYOZ3	myozenin 3
NAA20	N(alpha)-acetyltransferase 20, NatB catalytic subunit
NAMPT	nicotinamide phosphoribosyltransferase
NLGN2	neuroligin 2
NMI	N-myc (and STAT) interactor
Nrg1	neuregulin 1
NT5C3A	5'-nucleotidase, cytosolic IIIA
NUMA1	nuclear mitotic apparatus protein 1
OAS1	2'-5'-oligoadenylate synthetase 1, 40/46kDa
OAS2	2'-5'-oligoadenylate synthetase 2, 69/71kDa
OAS3	2'-5'-oligoadenylate synthetase 3, 100kDa
Oas1b	2'-5' oligoadenylate synthetase 1B
Oasl2	2'-5' oligoadenylate synthetase-like 2
OASL	2'-5'-oligoadenylate synthetase-like
Olfr56	olfactory receptor 56
OPCML	opioid binding protein/cell adhesion molecule-like
PABPC4	poly(A) binding protein, cytoplasmic 4 (inducible form)
PARP9	poly (ADP-ribose) polymerase family, member 9
PARP11	poly (ADP-ribose) polymerase family, member 11
PARP14	poly (ADP-ribose) polymerase family, member 14
PCGF5	polycomb group ring finger 5
PDE7B	phosphodiesterase 7B
PDGFC	platelet derived growth factor C
Phf11a/Phf11b	PHD finger protein 11A
PIK3AP1	phosphoinositide-3-kinase adaptor protein 1
PLEC	plectin
PML	promyelocytic leukaemia
PNP	purine nucleoside phosphorylase
POU3F1	POU class 3 homeobox 1

PPA1	pyrophosphatase (inorganic) 1
PRF1	perforin 1 (pore forming protein)
Prm1	protamine 1
RHBG	Rh family, B glycoprotein (gene/pseudogene)
RILPL1	Rab interacting lysosomal protein-like 1
Rnf213	ring finger protein 213
RSAD2	radical S-adenosyl methionine domain containing 2
RXRA	retinoid X receptor, alpha
SAMD9L	sterile alpha motif domain containing 9-like
SASS6	spindle assembly 6 homolog (C. elegans)
SCT	secretin
Serpina3g (includes others)	serine (or cysteine) peptidase inhibitor, clade A, member 3G
SFMBT2	Scm-like with 4 mbt domains 2
SGCB	sarcoglycan, beta (43kDa dystrophin-associated glycoprotein)
SLC15A3	solute carrier family 15, member 3
SLC4A11	solute carrier family 4, sodium borate transporter, member 11
Slfn1	schlafen 1
SLFN5	schlafen family member 5
SLFN12L	schlafen family member 12-like
SOAT2	sterol O-acyltransferase 2
SOCS1	suppressor of cytokine signalling 1
SP110	SP110 nuclear body protein
SPA17	sperm autoantigenic protein 17
SPON1	spondin 1, extracellular matrix protein
SPPL2A	signal peptide peptidase like 2A
STAT1	signal transducer and activator of transcription 1, 91kDa
STAT2	signal transducer and activator of transcription 2, 113kDa
STAU2	staufen double-stranded RNA binding protein 2
STX6	syntaxin 6
STX11	syntaxin 11
STXBP3	syntaxin binding protein 3
TDRD7	tudor domain containing 7
Tgtp1/Tgtp2	T cell specific GTPase 1
TKTL1	transketolase-like 1
TMEM171	transmembrane protein 171
TMEM106A	transmembrane protein 106A
TNFSF8	tumour necrosis factor (ligand) superfamily, member 8
TNFSF15	tumour necrosis factor (ligand) superfamily, member 15
TNP2	transition protein 2 (during histone to protamine replacement)
TOR1AIP2	torsin A interacting protein 2
TOR3A	torsin family 3, member A
TPST1	tyrosylprotein sulfotransferase 1
TREML2	triggering receptor expressed on myeloid cells-like 2
Trim30a/Trim30d	tripartite motif-containing 30A
UACA	uveal autoantigen with coiled-coil domains and ankyrin repeats
UBE2L6	ubiquitin-conjugating enzyme E2L 6
USP18	ubiquitin specific peptidase 18
USP25	ubiquitin specific peptidase 25
VCAN	versican
XAF1	XIAP associated factor 1
XCL1	chemokine (C motif) ligand 1
XDH	xanthine dehydrogenase
ZBP1	Z-DNA binding protein 1
ZNFX1	zinc finger, NFX1-type containing 1
